# Replicating RNA vaccination elicits an unexpected immune response that efficiently protects mice against lethal Crimean-Congo hemorrhagic fever virus challenge

**DOI:** 10.1016/j.ebiom.2022.104188

**Published:** 2022-07-27

**Authors:** Shanna S. Leventhal, Kimberly Meade-White, Deepashri Rao, Elaine Haddock, Jacqueline Leung, Dana Scott, Jacob Archer, Samantha Randall, Jesse H. Erasmus, Heinz Feldmann, David W. Hawman

**Affiliations:** a Laboratory of Virology, Division of Intramural Research, National Institute of Allergy and Infectious Diseases, National Institutes of Health, Rocky Mountain Laboratories, Hamilton, MT 59840, USA; b Research Technologies Branch, Division of Intramural Research, National Institutes of Allergy and Infectious Diseases, National Institutes of Health, Rocky Mountain Laboratories, Hamilton, MT, USA; c Rocky Mountain Veterinary Branch, Division of Intramural Research, National Institutes of Allergy and Infectious Diseases, National Institutes of Health, Rocky Mountain Laboratories, Hamilton, MT, USA; d HDT Bio, Seattle, WA 98102, USA; e Department of Microbiology, University of Washington School of Medicine, Seattle, WA 98109, USA

**Keywords:** Crimean-Congo hemorrhagic fever, CCHFV, Vaccine, RNA vaccine, Mouse

## Abstract

**Background:**

Crimean-Congo hemorrhagic fever virus is the cause of a severe hemorrhagic fever with cases reported throughout a wide-geographic region. Spread by the bite of infected ticks, contact with infected livestock or in the health care setting, disease begins as a non-specific febrile illness that can rapidly progress to hemorrhagic manifestations. Currently, there are no approved vaccines and antivirals such as ribavirin have unclear efficacy. Thus treatment is mostly limited to supportive care.

**Methods:**

In this report we evaluated an alphavirus-based replicon RNA vaccine expressing either the CCHFV nucleoprotein or glycoprotein precursor in a stringent, heterologous lethal challenge mouse model.

**Findings:**

Vaccination with the RNA expressing the nucleoprotein alone could confer complete protection against clinical disease, but vaccination with a combination of both the nucleoprotein and glycoprotein precursor afforded robust protection against disease and viral replication. Protection from lethal challenge required as little as a single immunization with 100ng of RNA. Unexpectedly, analysis of the immune responses elicited by the vaccine components showed that vaccination resulted in antibodies against the internal viral nucleoprotein and cellular immunity against the virion-exposed glycoproteins.

**Interpretation:**

Cumulatively this vaccine conferred robust protection against Crimean-Congo hemorrhagic fever virus and supports continued development of this vaccine candidate.

**Funding:**

This research was supported by the Intramural Research Program of the NIAID/NIH and HDT Bio.

**Research in context**

**Evidence before this study**

Crimean-Congo hemorrhagic fever virus (CCHFV) is the cause of a severe febrile illness with case fatality rates of up to 30%. CCHFV is spread by the bite of infected ticks, handling of infected livestock or in the health care setting. CCHF begins as a non-specific febrile illness and can progress to hemorrhagic disease. There are no licensed vaccines nor CCHFV-specific therapies, and intervention is mostly limited to supportive care. Due to the expanding range of the tick-vector, no vaccines and limited treatment options, the World Health Organization lists CCHFV as a priority pathogen for vaccine development. Furthermore, although several vaccine candidates have been evaluated in pre-clinical models, the correlates of vaccine-mediated protection are unknown.

**Added value of this study**

We have developed a self-replicating RNA vaccine for CCHFV using a Venezuelan Equine Encephalitis Virus (VEEV) replicon and cationic nanocarrier delivery system. We evaluated RNAs expressing either the CCHFV nucleoprotein (NP) or the CCHFV glycoprotein precursor (GPC). Although the glycoproteins are exposed on the virion surface, substantial genetic diversity exists in these proteins across CCHFV strains and incomplete protection when the vaccine and challenge strain of CCHFV are mismatched has been reported in other vaccine platforms. In contrast, the NP is highly conserved but is not exposed on the cell nor virion surface. In vaccinated mice we found that the NP-expressing vaccine elicited robust but non-neutralizing antibodies while unexpectedly, the GPC-expressing vaccine elicited mainly a CD8 T-cell response. Challenge of vaccinated mice with a strain of CCHFV highly divergent from the vaccine antigens demonstrated that the NP-expressing vaccine alone could confer complete protection against disease. Surprisingly, the GPC-expressing vaccine failed to protect, however, vaccinating mice with both the NP- and GPC-expressing vaccines conferred greatest control of viral replication. We further show that a low-dose prime-only vaccination confers complete protection against disease, even following high-dose challenge, demonstrating the utility of this vaccine in endemic regions with poor access to health care.

Lastly, to identify the correlates of vaccine-mediated protection we evaluated vaccination of mice lacking humoral immunity or depleted mice of cellular immunity at time of CCHFV challenge. We show that humoral immunity is essential to vaccine-mediated protection as most vaccinated mice lacking B-cells succumbed to disease. However, our data also show that cellular immunity may contribute to control of viral loads in key target tissues.

**Implications of all the available evidence**

Cumulatively, our data unexpectedly show that non-neutralizing antibody responses directed against an internal viral protein and cellular immunity to virion-exposed proteins confer robust control of CCHFV infection. These findings demonstrate that this vaccine is highly immunogenic, conferring robust immunity even after a single, low dose shot and also identify a key role of non-neutralizing antibodies in protection against CCHFV. Intriguingly, CCHFV-infection of naive humans typically results in early non-neutralizing antibody responses against the viral NP. Thus, our findings that NP-directed immunity can confer robust protection may further our understanding of how host responses can control the viral infection even in immunologically naïve patients. Overall, our data support continued development of this vaccine candidate to prevent the substantial morbidity and mortality in CCHFV-infected humans.

Alt-text: Unlabelled box

## Introduction

Crimean-Congo hemorrhagic fever virus (CCHFV) is a tick-borne bunyavirus endemic in Southern and Eastern Europe, Africa, the Middle East, and Asia.^1^ Geographically, case distribution is consistent with the range of *Hyalomma* genus ticks, the main reservoir of CCHFV, and is likely to expand due to climate change. Humans may be infected from tick bites, through contact with infected animals or animal tissues.^1^ Nosocomial human-to-human transmission has also been described primarily for healthcare workers.^2^ Initial symptoms of CCHF include acute onset of a non-specific febrile illness consisting of sudden fever, myalgia, diarrhea, nausea, and vomiting.^1^ The hemorrhagic phase is characterized by large areas of severe bruising and uncontrolled bleeding throughout the body; among hospitalized patients, case fatality rates have ranged from 9-50%.^1^ As there is no widely available and efficacious vaccine or therapeutic, the World Health Organization lists CCHFV as a high priority pathogen for development of antiviral countermeasures.

CCHFV is a tri-segmented, negative sense, RNA bunyavirus in family *Nairoviridae* with small (S) encoding the nucleoprotein (NP), medium (M) encoding the glycoprotein precursor (GPC), and large (L) genomic segments encoding the viral RNA-dependent RNA-polymerase.^3^ As the correlates of vaccine-mediated protection are unknown, vaccine development has focused on the GPC and NP antigens and several vaccine candidates consisting of either antigen alone or a dual-antigen approach have been evaluated in animal models with varied results.^4^ While the viral glycoproteins are a major target of both neutralizing and non-neutralizing antibodies, NP can also be targeted by antibodies, both NP and GPC can induce T-cell responses, and the NP is more conserved among geographically diverse CCHFV strains. These attributes make both the GPC and NP antigens attractive targets for vaccines.

Here, we evaluated a novel alphavirus-based replicon RNA (repRNA) vaccine expressing either the CCHFV NP, GPC or both. Replicon based RNA vaccines have been explored for a variety of viral pathogens^5^ and their ability to drive high levels of protein expression, stimulation of host innate immunity and mimicry of an authentic viral infection, make them promising vaccine platforms. We have previously reported on repRNAs delivered via cationic nanocarrier (CNC) for SARS-CoV-2^6^, ^7^ and this platform is in several clinical trials for COVID-19.^8^ We found that a low-dose, single-shot vaccination with CCHFV-specific repRNAs could elicit robust CCHFV-specific B- and CD8^+^ T-cell responses and protect mice lethally challenged with a highly divergent strain of CCHFV. This robust protection was mediated largely through the CCHFV NP antigen although inclusion of the GPC antigen significantly improved viral control. Studies in immune deficient mice demonstrated that vaccine mediated protection from clinical disease required CCHFV-specific humoral responses. Cumulatively this vaccine elicited robust protective immunity in a stringent lethal mouse model and supports continued development of this vaccine for CCHFV.

## Methods

### Ethics

All work with infectious CCHFV was done following guidelines put forth by the Institutional Biosafety Committee (IBC) in biocontainment level 4 at the Rocky Mountain Laboratories, NIAD, NIH, Hamilton, MT. Animal work was approved by the Rocky Mountain Laboratories Institutional Animal Care and Use Committee (protocol #2020-76) in accordance with recommendations by the Guide for the Care and Use of Laboratory Animals of the National Institutes of Health, the Office of Animal Welfare, the United States Department of Agriculture in an association for Assessment and Accreditation of Laboratory Animal Care-Accredited Facility. Mice were housed in HEPA-filter cage systems enriched with nesting material and commercial food and water available ad libitum

### Vaccine

repRNAs were constructed using standard cloning techniques and sequences for NP and a codon-optimized GPC from CCHFV strain Hoti (accession #s MH483984 and MH483985, respectively) were fused to a 3’ V5- or HA-epitope tag, respectively, to facilitate protein expression studies. A generalized schematic of the repRNAs is shown in Supplemental Figure 1. Vaccine RNA was synthesized *in vitro* and was complexed to CNC as described previously.^6^

### Mice, vaccinations, and infection

Wild-type C57BL6/J (stock #00664) mice or μMT mice on the C57BL6/J background (stock #002288) were purchased from Jackson Laboratories. Male and female mice were used for all studies and were all approximately 8-weeks of age at time of vaccination. Mice were vaccinated via a single 50μL intramuscular injection to the hind limb. Vaccination appeared well tolerated and no adverse events attributable to the vaccine were observed during the studies. At time of CCHFV challenge, mice received 2.5mg MAR1-5A3 (Leinco) and indicated dose of CCHFV strain UG3010 via intraperitoneal injections. Body temperature was recorded using a Unified Information Devices telemetry system and UID Mouse Matrix reader plates. Mice were implanted with telemetry transponders (UCT-2112, UID) via subcutaneous implantation and mice allowed to recover for at least one-week prior to CCHFV challenge. Data was recorded continuously with a zone interval of 250ms, 2 cycles per series and a 1s series delay. Data reported as mean of readings collected during 12-h intervals corresponding to vivarium light-dark cycles.

### Viral stock

CCHFV strain UG3010 was originally provided by Eric Bergeron, Centers for Disease Control and Prevention. On site, UG3010 was grown and titrated on SW13 cells in L-15 (ATCC) supplemented with 2% fetal bovine serum, 2 mM L-glutamine, 50 µg/mL penicillin, and 50 µg/mL streptomycin. Virus stock was sequenced via Illumina-based sequencing to confirm identity and exclude contamination. Sequence identity confirmed compared to Genbank accession numbers DQ211650, DQ211637, DQ211624.

### In vivo depletions

Control mice were treated with 200 µg of rat IgG2b isotype (clone 1-2) while T-cell depletion mice were treated with 200μg α-CD4 (clone GK1.5), α-CD8 (clone 2.43), or both diluted in neutral pH sterile phosphate buffered saline (PBS) and administered via 100uL intraperitoneal (IP) injections on day −5 and -2 relative to CCHFV-challenge. On day 0, a group of mice were euthanized for evaluation of depletion efficacy via flow cytometry.

### ELISA

An in-house ELISA using whole CCHFV Hoti antigen was used to quantify CCHFV-specific antibodies as previously described.^9^

### Western blot and immunofluorescence assay

BHK21 cells were transfected with repNP or repGPC RNA using TransIT-mRNA Transfection Kit (Mirus) and incubated overnight. Next day, cell lysate harvested using Thermofisher RIPA Lysis and Extraction Buffer and Roche c0mplete protease-inhibitor tablets and recommended protocols. Samples were mixed 1:1 with Laemlli buffer (10% SDS, 0.1M DTT), heated for 10min at 99˚C, briefly incubated on ice, and loaded onto a 12-well Biorad Criterion TGX precast Gel 10% alongside Biorad Precision Plus Protein Dual Color Standards. After transfer to nitrocellulose membrane, blots stained with primary antibody mouse anti-V5 (Invitrogen) for NP or mouse anti-HA (Thermofisher) for GPC, washed and incubated with anti-mouse HRP (Jackson Immunoresearch). Blot was washed and imaged using supersignal west pico PLUS chemiluminescent substrate (Fisher Scientific) and Proteinsimple FluorChem E Imager. For IFA, cells were washed, fixed in 1% PFA in DPBS for 10 minutes, permeabilized and incubated with the respective mouse anti-V5 or mouse anti-HA antibody. Cells were then washed, incubated with secondary Goat anti-Mouse IgG (H+L) conjugated to Alexa Fluor 488 (Thermofisher), washed and imaged on Biorad ZOE fluorescent cell imager.

*IFNγ ELISpot.* An IFNγ ELISpot to evaluate CCHFV-specific T-cell responses was performed as previously described^9^ using a mouse single-color IFNγ kit (Cellular Technologies Limited). Briefly, fresh splenocytes were diluted in CTL-Test medium, 300,000 - 500,000 cells per well were plated and stimulated with 1 μg/ml each peptide of 15-mer peptides overlapping by 11 amino acids derived from the Hoti NP or GPC sequence. Peptides were synthesized (Genscript) and dissolved in dimethyl sulfoxide (DMSO; Hybrimax grade [Sigma]) and pooled with 19 to 30 peptides per pool. As positive control, cells were stimulated with concanavalin A (Life Technologies) while negative controls were stimulated with CTL-Test medium containing DMSO vehicle alone. Cells were incubated for 24 h at 37°C and developed according to manufacturer protocols. Spots were counted and analyzed using an S6 Universal Analyzer (CTL) and SFCs normalized to 10^6^ splenocytes.

### Quantitative reverse-transcription PCR (qRT-PCR)

RNA was extracted from blood and tissue samples using Qiagen Qiamp viral RNA-mini-isolation kit and Qiagen RNeasy mini-isolation kit, respectively, and provided protocols. Viral RNA was quantified using Qiagen Quantifast one-step qRT-PCR master mix and primers and probe specific for the CCHFV NP (Forward: 5’ AAAATGAAGAAGGCACTCCTGAG 3’, Reverse: 5’GCAGACACCCATTTCACTGATTCT 3’ and probe 5’ CCAATGAAGTGGGGAAAGAA 3’ with a 5’ 6-FAM, and an internal and 3’ quencher). Primers and probes were purchased from IDT. Reaction was run on a Quantstudio 5 RT-PCR system (ThermoFisher) with cycling conditions as follows: initial hold of 50°C 10 min, initial denaturation of 95°C 5 min, and 40 cycles of 95°C 15s, 50°C for 20s and 72°C for 1s. *In vitro* transcribed RNA standards with known copy number were prepared in house, diluted, and run alongside samples for quantification. No template controls were included in each run.

### Median tissue culture infectious dose (TCID_50_) assay

Infectious virus was quantified on SW13 cells. SW13 cells were plated in 96-well tissue culture plates in L-15 media supplemented with 10% fetal bovine serum, 2mM L-glutamine, 50 U/mL penicillin and 50 µg/mL streptomycin. Blood was initially diluted 1:10 in PBS without Ca2+/Mg2+ before subsequent 1:10 dilutions in L-15 media supplemented with 2% fetal bovine serum, 2mM L-glutamine, 50 U/mL penicillin and 50 µg/mL streptomycin (dilution media). Lung and spleen samples were weighed and then homogenized in 1mL dilution media with sterile bead before subsequent 1:10 dilutions as with blood. 100uL of each dilution was transferred to SW13 cells. SW13 with sample were incubated at 37°C for 5 days before CPE was read. Lung and spleen titers normalized to mg of tissue. Negative samples were set to 0.5 Log(TCID50/mg). TCID50 was calculated using the Reed & Muench method.

### Neutralization assay

SW13 cells were plated in L-15 Media (ATCC) supplemented with 2% fetal bovine serum, 2 mM L-glutamine, 50 µg/mL penicillin, and 50 µg/mL streptomycin for 80–90% confluency at time of assay on 96-well tissue culture plates. Sera was inactivated at 56 °C for 30 minutes and serially diluted 1:2 in triplicate starting with a 1:10 dilution in infection media (LT-15 as above with 2% FBS). CCHFV Hoti was diluted to contain 120 TCID_50_ and added 1:1 to sera dilutions and incubated at 37 °C for one hour after which 100uL sera-virus mixture was deposited onto SW13 cells and incubated at 37 °C. Serum toxicity was checked after 24h and CPE on Day 5.

### Histology

Tissues were fixed in 10% Neutral Buffered Formalin x2 changes, for a minimum of 7 days. Tissues were placed in cassettes and processed with a Sakura VIP-6 Tissue Tek, on a 12-h automated schedule, using a graded series of ethanol, xylene, and ParaPlast Extra. Embedded tissues are sectioned at 5 µm and dried overnight at 42°C prior to staining. Sections were stained with hematoxylin and eosin or specific anti-CCHFV immunoreactivity was detected using a rabbit anti-CCHFV N protein antibody (IBT Bioservices cat#04-0011) at a 1:2000 dilution. The secondary antibody was the ImPress VR anti-rabbit IgG polymer (Vector Laboratories cat# MP-6401). The tissues were processed for immunohistochemistry using the Discovery Ultra automated stainer (Ventana Medical Systems) utilizing the ChromoMap DAB kit (Roche Tissue Diagnostics cat#760-159). Scoring was performed by a pathologist blinded to study groups.

### Statistics

Indicated statistical tests were performed using GraphPad Prism 9. Sample size determination was determined from our experience with CCHFV mouse models. Animals were randomly assigned to study groups. Pathologist was blinded to study groups. Remaining research staff were not blinded to study groups. One sham-vaccinated animal was excluded from the prime-only vaccination survival analyses. This animal was excluded as it showed no weight loss upon infection and ELISA analysis of serum collected at day 14 PI showed no seroconversion to CCHFV. No other animals were excluded from analysis.

### Role of funding

Funders had no input on study design, data collection, interpretation, data analysis, writing of report or decision to publish.

## Results

### repNP, repGPC, and repNP + repGPC RNA vaccinations induce significant B and T cell responses

**Figure 1:**
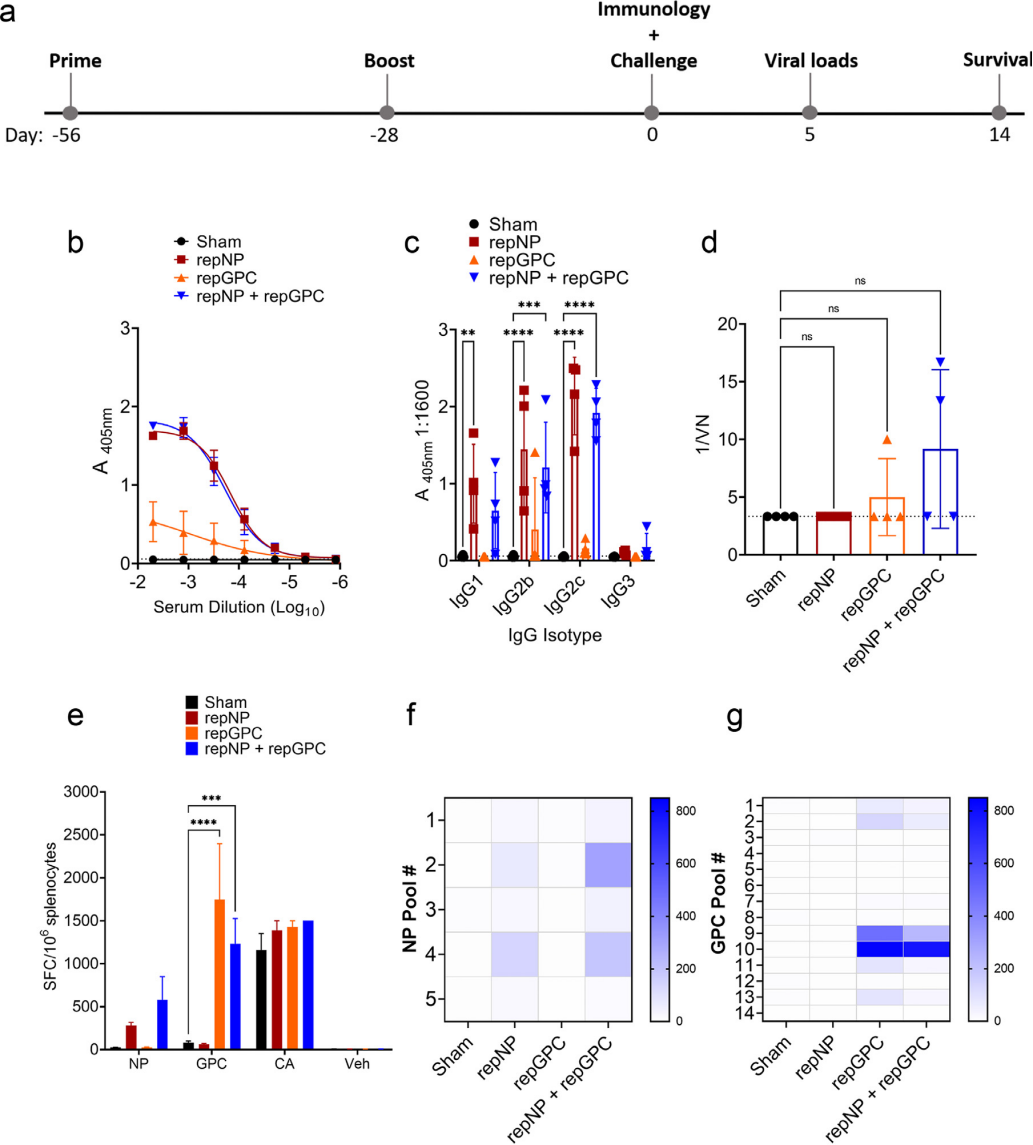
**repRNA vaccination elicits humoral and cellular immunity to CCHFV.** WT C57BL6/J mice were given (a) prime boost vaccinations at days -56 and -28 relative to CCHFV challenge on day 0. On day 0 groups of four mice vaccinated with 2.5μg (sham, repNP, repGPC) or 5μg (repNP + repGPC) of RNA were evaluated for immune responses to CCHFV. CCHFV-specific antibody was measured by whole-virion ELISA for total IgG (b) or specific isotypes (c). Dashed line indicates background absorbance of wells that received no serum. Serum neutralization activity was measured by a microneutralization assay against infectious CCHFV strain Hoti (d). Dashed line indicates limit of detection and statistical significance calculated using a one-way ANOVA with Dunnett's multiple comparison test. CCHFV-specific T-cell responses were measured by IFNγ ELISpot (e – g). Cumulative SFCs against peptide pools spanning the entire NP or GPC, the mitogen concanavalin a (CA) or DMSO vehicle alone (veh) are shown. Statistical comparisons calculated using a two-way ANOVA with Dunnett's multiple comparison test (c – e) Heat maps showing the distribution of measured IFNγ SFCs against NP (e) or GPC (f) peptide pools. ns P > 0.05, *** P < 0.001. (b – e) Data shown as mean plus standard deviation.

We evaluated mice vaccinated with replicon RNAs expressing either the CCHFV strain Hoti NP (repNP) or GPC (repGPC) or mice immunized with both (repNP + repGPC). As control, sham vaccinated mice received a repRNA expressing the irrelevant green fluorescent protein (GFP). We confirmed protein expression of each antigen *in vitro* by both western blot and immunofluorescence, although expression of NP appeared more robust (Supplemental Figure 1). We next evaluated immunogenicity *in vivo* in WT C57BL6/J mice vaccinated with a prime-boost regimen (Figure 1a). Vaccinations were conducted in 4-week intervals with 2.5μg of each RNA, or 5μg total RNA for repNP + repGPC, complexed to CNC. Mice were vaccinated with an intramuscular (IM) injection. To evaluate systemic vaccine-induced immune responses, groups of mice were analyzed for homologous CCHFV-specific responses four-weeks after boosting by ELISA, immunofluorescence assay (IFA), virus neutralization and ELISpot. As measured by ELISA, four-weeks after their second immunization, mice vaccinated with repNP and repNP + repGPC had robust CCHFV-specific IgG titers compared to sham vaccinated animals that received a repRNA expressing GFP (Figure 1b). Consistent with repRNA mimicking an authentic viral infection,^10^^,^^11^ the CCHFV-specific IgG response was largely comprised of IgG2c, IgG2b and to a lesser degree IgG1 isotypes (Figure 1c). In contrast, repGPC vaccinated animals had low levels of CCHFV-specific IgG (Figure 1b & c) with only 1 of 4 evaluated animals having titers above background suggesting repGPC largely failed to elicit a humoral immune response. To confirm our ELISA findings, we performed an IFA on CCHFV-infected cells (Supplemental Figure 2). Pooled sera from repNP vaccinated mice robustly labeled intracellular antigen in CCHFV infected cells while sera from repGPC-only vaccinated mice also labeled CCHFV-infected cells, albeit with reduced intensity compared to sera from repNP animals (Supplemental Figure 2). Little reactivity was observed when sera from repNP or repGPC vaccinated animals was used to label non-permeabilized cells (Supplemental Figure 2J–L), consistent with the intracellular localization of these antigens.^12^, ^13^, ^14^ Further, homologous neutralizing titers in any vaccinated group were low, and the increases were not significant compared to sham-vaccinated mice (Figure 1 d). We next evaluated whether repNP and/or repGPC vaccination could elicit cellular immunity through IFNγ ELISpot with splenocytes stimulated by overlapping peptide pools spanning the entire CCHFV Hoti NP or GPC (Figure 1e–g). Cumulatively our ELISpot data showed that the rep-expressed NP antigen only weakly elicited a non-significant T-cell response against NP peptides compared to sham-vaccinated animals (Figure 1e). Similarly, repNP + repGPC vaccinated animals had no significant increase in spot-forming cells (SFCs) against the NP peptide pools (Figure 1e). In contrast, the rep-expressed GPC antigen elicited robust cellular immunity with repGPC and repNP + repGPC vaccinated animals having significant increases in IFNγ SFCs against the GPC peptide pools (Figure 1e). Against NP, the T-cell response in repNP and repNP + GPC vaccinated animals was directed against peptide pools 2 (aa 101 – 211) and 4 (aa 301 – 411) (Figure 1f). In repGPC vaccinated animals IFNγ T cell responses were directed towards GPC peptide pools 9 and 10 (Figure 1g) which span from aa 961 to 1211 comprising the NSm and N-terminal half of Gc (Supplemental Figure 1d). Cumulatively, our immunological analyses of repNP and repGPC vaccinated mice demonstrated that repNP vaccination primarily elicited a robust but non-neutralizing antibody response while repGPC elicited primarily cellular immunity against epitopes in the CCHFV NSm and Gc proteins.

### repNP and repNP + repGPC vaccinations protect against heterologous CCHFV challenge

**Figure 2:**
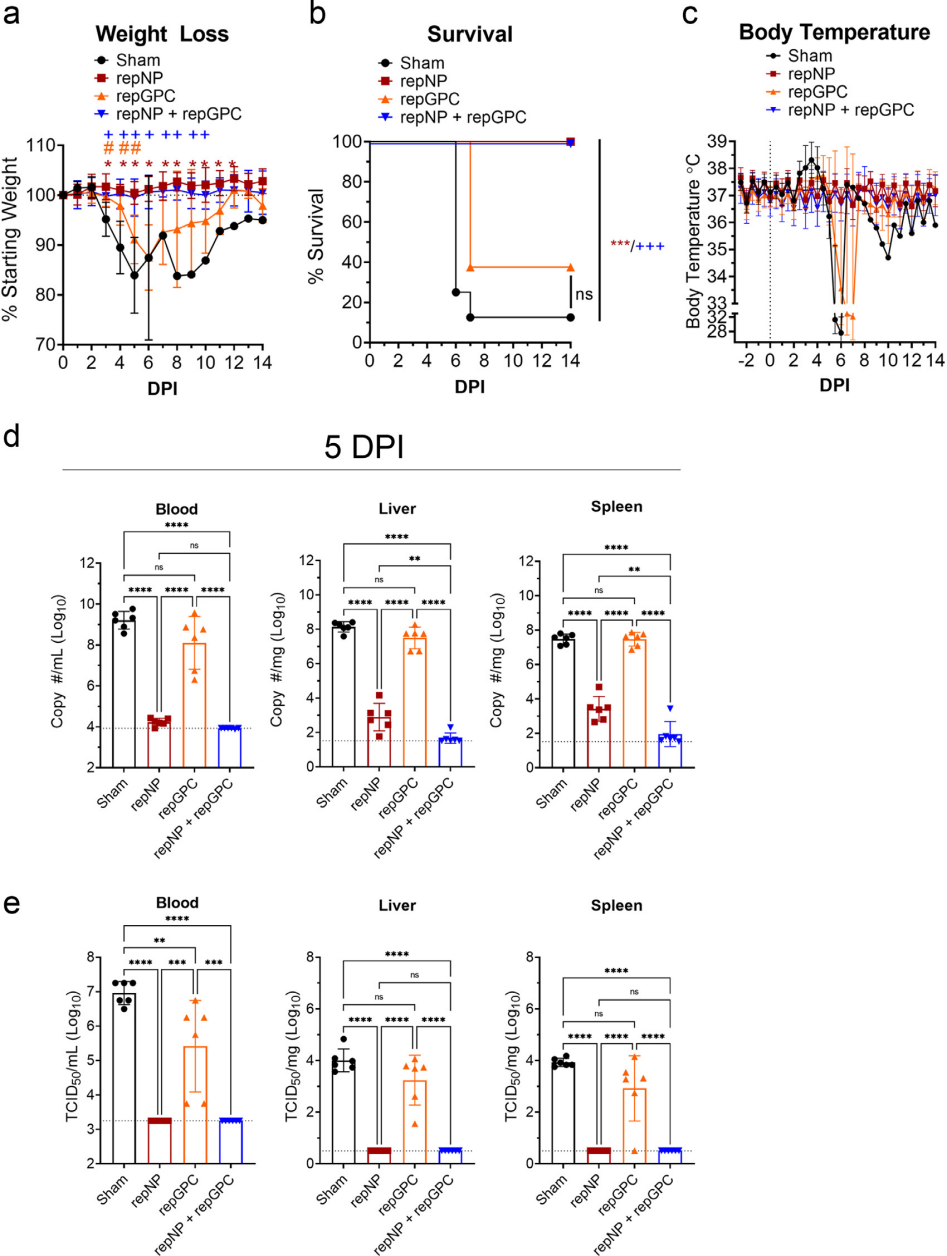
**repNP vaccination confers significant protection against CCHFV.** Groups of WT mice given indicated prime-boost vaccinations were treated with MAR1-5A3 to blockade type I IFN and infected with CCHFV strain UG3010. Mice were weighed daily (a), monitored for survival (b) and body temperature continuously monitored via telemetry system (c). N = 8 mice per group. Statistical comparisons were calculated using a two-way ANOVA with Dunnett's multiple comparison test (a) or Log-Rank test with Bonferonni's correction for multiple comparisons (b). Statistical significance compared to sham vaccinated animals is shown with symbols: * (repNP), # (repGPC) and + (repNP + repGPC). Viral loads in indicated tissues at day 5 p.i was quantified by qRT-PCR. Dashed line indicates limit of detection (d). N = 6 mice per group Statistical comparisons calculated using a one-way ANOVA with Tukey's multiple comparison test. ns P > 0.05, * P < 0.05, ** P < 0.01, *** P < 0.001, **** P < 0.0001. (a, c, d) Data shown as mean plus standard deviation.

We next evaluated the efficacy of these vaccines against lethal heterologous challenge with 100 TCID_50_ of CCHFV strain UG3010. Compared to the vaccine antigens derived from CCHFV strain Hoti, at the amino-acid level, CCHFV strain UG3010 differs by 4.5% in the NP, 25.6% across the whole GPC and 14% specifically in the Gn and Gc glycoproteins. (Supplemental Figure 1d). Thus, this mismatch between vaccine antigens and CCHFV challenge strain would provide a stringent model to test efficacy of repRNA vaccination. Mice were treated with the type I IFN receptor blockade antibody MAR1-5A3 at time of CCHFV challenge to block type I IFN signaling and render mice susceptible to lethal UG3010 infection.^15^^,^^16^ We used this transient type I IFN suppression CCHF model^15^ rather than vaccination of genetically type I IFN deficient mice (e.g. IFNAR^−/−^) to avoid confounding factors of type I IFN deficiency on development of immune responses to vaccination, particularly self-amplifying RNA vaccines.^17^^,^^18^ Most sham-vaccinated animals infected with UG3010 began losing weight on day 3 p.i., and exhibited hyperthermia followed by hypothermia and death by day 7 p.i. (Figure 2a–c). One sham-vaccinated animal exhibited delayed disease, not showing clinical disease until day 7 p.i., and ultimately survived the infection (Figure 2a–c). Over the 14-day challenge, both repNP and repNP + repGPC vaccinated animals had no evidence of clinical disease (Figure 2a - c) and all vaccinated animals survived the infection (P < 0.001) (Figure 2b). In contrast, repGPC vaccinated animals began to lose weight starting at day 4 p.i. (Figure 2a), had evidence of hypothermia beginning on day 5 (Figure 2c) and 5 of 8 repGPC vaccinated animals succumbed to the infection by day 7 p.i (Figure 2b). Surviving sham and repGPC vaccinated animals began recovering weight after days 9 and 6 p.i., respectively (Figure 2a). These data demonstrate that repNP vaccination could protect against clinical disease following lethal heterologous CCHFV challenge.

Consistently, viral loads as measured by qRT-PCR and TCID_50_ assay in the blood, liver, and spleen at day 5 p.i. showed that both repNP and repNP + repGPC vaccinated animals had significantly lower viral loads compared to either repGPC vaccinated or sham vaccinated animals (Figure 2d & e). Consistent with clinical disease and death in repGPC vaccinated animals, although repGPC vaccinated animals had slightly but significantly reduced infectious virus in the blood (Figure 2e), viral loads in the liver and spleen were similar to sham-vaccinated mice (Figure 2d & e) suggesting that repGPC vaccination alone could not control the CCHFV infection. By qRT-PCR, mice vaccinated with both repNP + repGPC had significantly lower viral loads in the liver and spleen compared to repNP alone (Figure 2d), suggesting that repGPC vaccination could contribute to controlling CCHFV replication when in combination with repNP. However, no infectious virus was detected in either group (Figure 2e) demonstrating that repNP alone conferred robust protection against viral replication.

**Figure 3:**
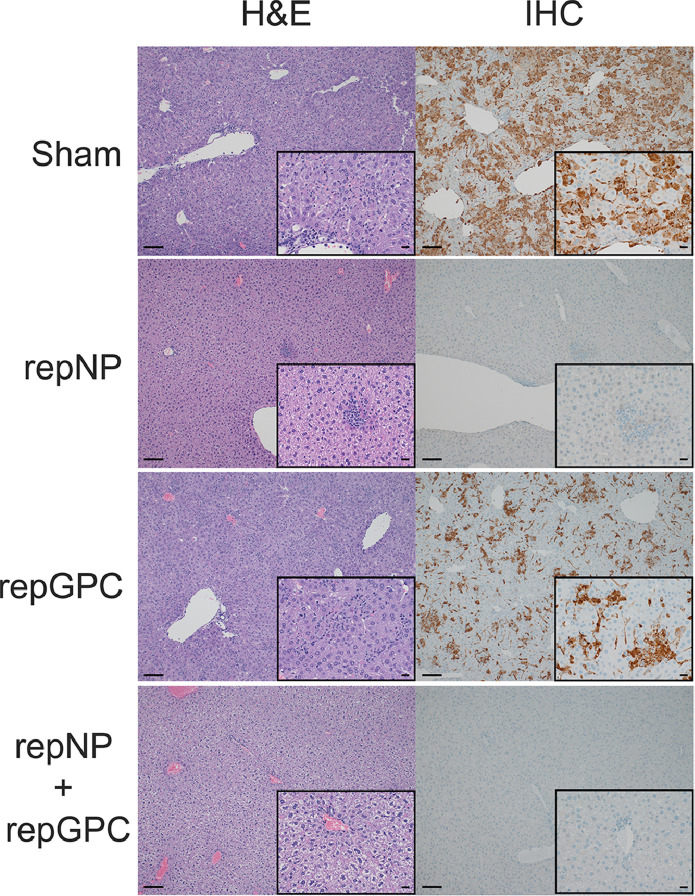
**repNP vaccination protects against liver pathology in CCHFV infected mice.** Groups of WT mice given indicated prime-boost vaccinations were treated with MAR1-5A3 to blockade type I IFN and infected with CCHFV strain UG3010. On day +5 p.i. mice were euthanized, liver collected and formalin fixed. Sections were H&E stained or probed for presence of viral antigen via immunohistochemistry (IHC). Representative images for each group are shown at 100x or 400x (inset) magnification and scale bars indicate 100μm or 20μm respectively. Complete findings are provided in Supplemental Table 1.

Consistent with our viral load data, histological analyses of formalin fixed liver and spleen sections from CCHFV-infected mice at day 5 p.i. showed little-to-no evidence of pathology nor viral antigen in repNP and repNP + repGPC vaccinated animals (Figure 3 and Supplemental Table 1). In contrast, sham vaccinated mice developed multifocal to coalescing coagulative necrosis of hepatocytes admixed with small numbers of viable and degenerative neutrophils and fewer macrophages. Remaining hepatocytes often demonstrated lipid-type vacuolar degeneration (Figure 3 and Supplemental Table 1). The spleens from these mice had extensive necrosis and loss of lymphocytes from the white pulp, as well as mild reticuloendothelial hyperplasia of the red pulp (Supplemental Table 1). Immunohistochemical analysis revealed viral antigen in the majority of hepatocytes, Kupffer cells, splenic reticuloendothelium and both hepatic and splenic endothelial cells (Figure 3 and Supplemental Table 1). Compared to sham-vaccinated animals, repGPC vaccinated animals had similar hepatic and splenic lesions but levels of viral antigen were reduced (Figure 3 and Supplemental Table 1). Together these data demonstrate that repNP provides robust protection against lethal CCHFV challenge.

### Dose-down prime-only and prime-boost NP + GPC vaccinations induce significant B and T cell responses

**Figure 4:**
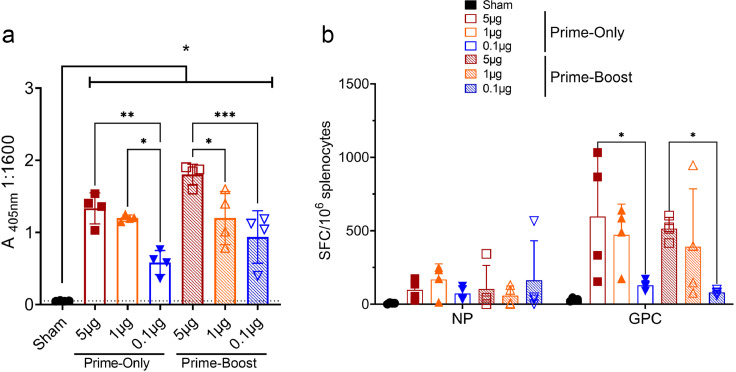
**Single-immunization with repRNA induces humoral and cellular immunity to CCHFV.** Groups of 4 WT mice were vaccinated with indicated cumulative doses of repNP + repGPC RNA in a prime-boost regimen as before or in a prime-only regimen. Four weeks after last vaccination, vaccine-induced immune responses to vaccination were measured by ELISA (a) or IFNγ ELISpot (b). CCHFV-specific IgG responses were measured by whole virion ELISA (a). Dashed line indicates background absorbance of wells receiving no serum. Statistical comparisons calculated using a two-way ANOVA using Tukey's multiple comparisons test. The summary P value of each vaccine group individually compared against sham-vaccinated animals is also shown. (b) CCHFV-specific T-cell responses were measured by IFNγ ELISpot and cumulative SFCs against the NP or GPC peptide pools is shown. Indicated statistical comparisons calculated using a two-way ANOVA with Tukey's multiple comparisons test. ns P > 0.05, * P < 0.05, ** P < 0.01, *** P < 0.001. Data shown as mean plus standard deviation.

Although both repNP and repNP + repGPC vaccinations conferred protection against clinical disease following CCHFV infection, repNP + repGPC vaccination conferred the greatest control over viral replication. Thus we elected to continue evaluation of repNP + repGPC in dose-down studies and whether prime-boost vaccination was necessary for protection. We evaluated mice vaccinated with 5μg, 1μg, and 0.1μg total repNP + repGPC RNA complexed to CNC in both prime-only and prime-boost schedules. As before, prime-boost mice were vaccinated 4 weeks apart or just 4 weeks prior to evaluation of the immunological response to vaccination. All groups had significantly increased IgG titers compared to sham vaccinated animals (Figure 4a) demonstrating that a single immunization with as low as 0.1μg of repRNA (0.05μg each RNA) could elicit significant CCHFV-specific IgG responses. In prime-only animals, the 5μg and 1μg groups had similar titers while the titer for 0.1μg animals was significantly decreased compared to both higher dose groups (Figure 4a). In prime-boost animals, the 5μg group had significantly higher titers compared to 1μg and 0.1μg groups (Figure 4a). Interestingly, when comparing mice vaccinated with similar doses in prime-only to prime-boost regimens there was no significant (P > 0.05) difference in CCHF-specific IgG (Figure 4a) indicating that boosting at 4 weeks did not significantly increase antibody responses. Similar to our previous data, cellular immunity as measured by IFNγ ELISpot against NP was weak and compared to sham vaccinated animals, no significant increases in SFCs were observed when splenocytes from any vaccination group were stimulated with NP peptides (Figure 4b). In contrast, compared to sham vaccinated animals, significant (two-way ANOVA, P < 0.05) responses to GPC were observed in the 5μg and 1μg prime-only and 5μg prime-boost groups (Figure 4b). Similar to our ELISA data, there was no significant difference (two-way ANOVA, P > 0.05) between prime-only and prime-boost ELISpot responses further suggesting that boosting at 4-weeks may not increase immunological response to the vaccine. Together, these data demonstrate that a single vaccination with as low as 50ng of each repRNA is sufficient to confer CCHFV-specific immunity.

### Low dose repNP + repGPC RNA vaccinations protect against heterologous CCHFV challenge in both prime-only and prime-boost vaccine schedules

**Figure 5:**
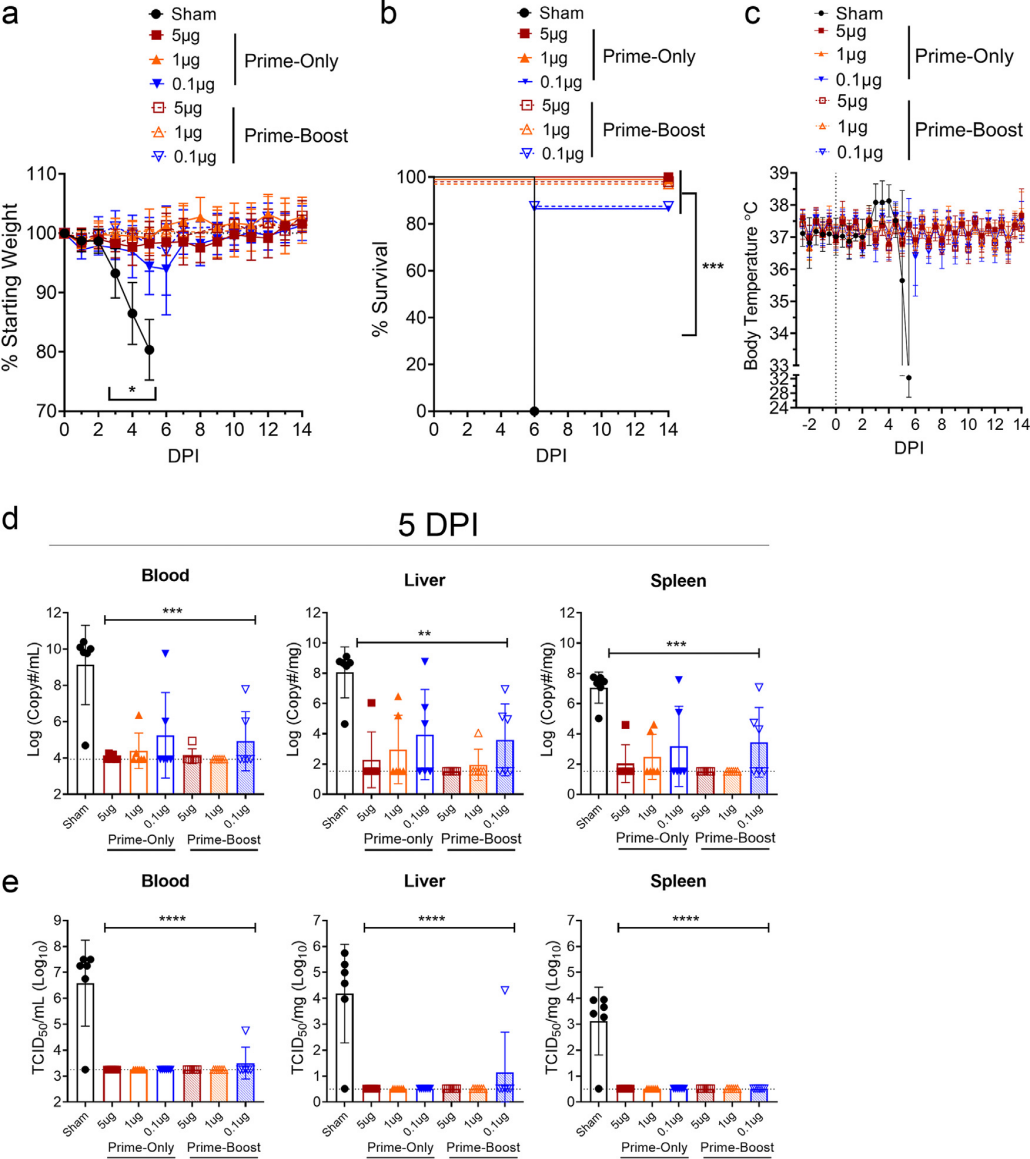
**Prime-only vaccination protects against CCHFV challenge.** Groups of WT mice receiving indicated vaccinations were treated with MAR1-5A3 to abolish type I IFN signaling and challenged with CCHFV strain UG3010. Mice were weighed daily (a), monitored for survival (b) and body temperature monitored continuously via telemetry system (c). N = 8 mice per group except for sham-vaccinated telemetry data where N = 6. Statistical comparison to sham-vaccinated mice performed using a two-way ANOVA with Dunnett's multiple comparisons test or log-rank test with Bonferroni's correction for multiple comparisons (b). At day 5 p.i., viral loads in indicated tissues were quantified by qRT-PCR (d) or TCID_50_ assay (e) and statistical comparison between sham vaccinated mice and repRNA-vaccinated mice calculated using one-way ANOVA with Dunnett's multiple comparisons test. Statistical comparisons between sham-vaccinated and every repNP-vaccinated groups were significant. N = 6 per group. ns P >0.05, * P < 0.05, ** P < 0.01, *** P <0.001, **** P < 0.0001. (a, c, d, e) Data shown as mean plus standard deviation.

To further investigate the relationship between RNA dose, dosing schedule and protective efficacy of vaccination against CCHFV challenge, 5μg, 1μg, and 0.1μg vaccinated groups of mice were treated with MAR1-5A3 and challenged with 100 TCID_50_ of CCHFV UG3010 as before. Mice vaccinated with 5μg or 1μg of repRNA in either prime-only and prime-boost regimens had no signs of clinical disease and 100% of mice survived the infection (log-rank test, p < 0.001) (Figure 5a–c). Although the 0.1μg prime-only group had a slight decrease in body weight around days 5 and 6 p.i. animals recovered to ∼100% initial starting weight by day 7 p.i. (Figure 5a). One mouse in each of the groups vaccinated with 0.1μg in either prime-only or prime-boost regimens succumbed on day 6 p.i. (Figure 5b) but survival was significantly improved compared to sham-vaccinated animals (log-rank test, p < 0.001). At day 5 p.i., as measured by both qRT-PCR and TCID_50_ assay, all repRNA vaccinated animals had significantly decreased viral loads in the blood, liver, and spleen (Figure 5d & e) demonstrating that a single low-dose immunization could confer robust control of CCHFV replication. Overall, most repRNA-vaccinated animals had no detectable viral RNA or infectious virus in the blood, liver, and spleen (Figure 5d) and infectious virus was only detected in the blood and spleen of one animal in the prime-boost 0.1μg group (Figure 5e). Cumulatively, these data show that a single vaccination with as little as 0.1μg repRNA confers near complete protection against CCHFV challenge.

To further evaluate control of CCHFV infection, we evaluated serum at day 14 p.i. of surviving mice to determine if CCHFV challenge of vaccinated mice resulted in an anamnestic antibody response. Consistent with mild clinical disease and trending increases in viral RNA loads, the 0.1µg prime-only and prime-boost groups had a significant anamnestic response evidenced by increased CCHFV-specific IgG response compared to day 0 (Supplemental Figure 3). In contrast, and consistent with robust control of the CCHFV challenge, neither the 5µg nor the 1µg groups experienced a significant increase in CCHFV-specific IgG at day 14 p.i. compared to day 0 (Supplemental Figure 3).

### Prime-only repNP vaccination protects against high-dose CCHFV challenge

We next investigated whether prime-only protection was achievable with just repNP. A cohort of mice were sham vaccinated or received a single vaccination with 1μg repNP. Four weeks later immune responses to the vaccine were evaluated via ELISA and ELIspot (Supplemental Figure 4a & b) and a single immunization with repNP was immunogenic with significant CCHFV-specific IgG and CCHFV-specific T-cell responses, albeit the T-cell responses were low (Supplemental Figure 4a & b). To stringently evaluate vaccine protection, mice were challenged with a high dose of CCHFV UG3010 (10,000 TCID_50_). Mice vaccinated with a single dose of repNP were significantly protected from disease showing little-to-no weight loss nor death (Supplemental Figure 4c & d). Two mice in the repNP-vaccinated group showed transient weight loss on days 6 and 7 p.i. (peak weight loss 7 – 11%) (Supplemental Figure 4c) that was also associated with transient hypothermia (Supplemental Figure 4e) suggesting protection was incomplete in these mice. Nevertheless, all mice in the repNP-vaccinated group survived. Consistent with protection from clinical disease, compared to sham-vaccinated animals, as measured by both qRT-PCR and TCID_50_ assay repNP-vaccinated animals had significantly reduced viral loads in the blood, liver and spleen at day 4 p.i. (Supplemental Figure 4f & g). Together these data demonstrate that a single immunization with repNP alone can protect against a high-dose CCHFV challenge.

### Humoral responses are sufficient to confer protection against lethal CCHFV challenge after single-dose repNP + repGPC vaccination

**Figure 6:**
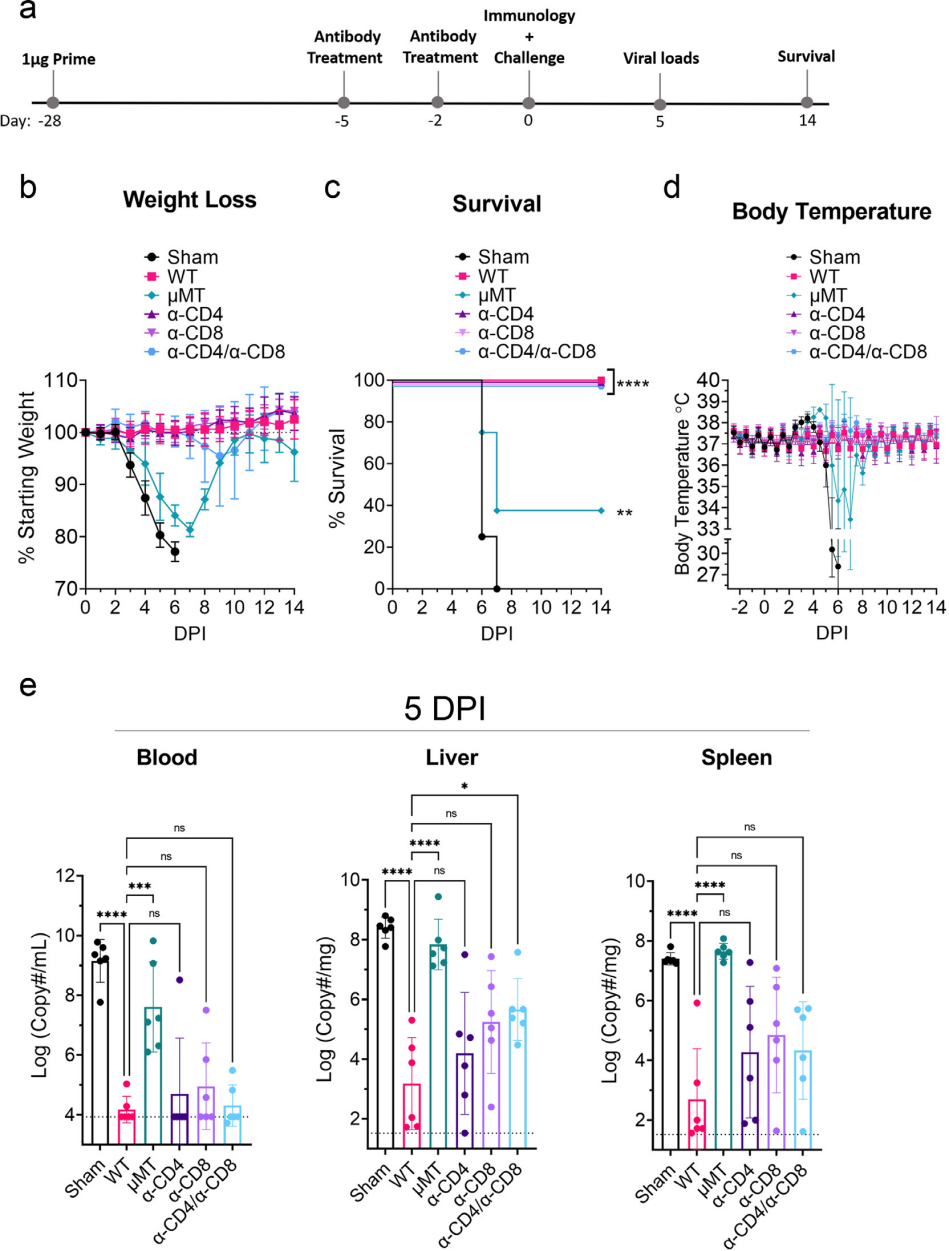
**Humoral immunity is required for protection from CCHFV challenge.** Groups of WT or B-cell deficient μMT mice were vaccinated with 1μg of repNP + repGPC RNA or sham vaccinated in a prime-only vaccination four weeks prior to CCHFV challenge (a). On days -5 and -2 relative to CCHFV challenge, groups of repNP + repGPC vaccinated mice were treated with an isotype control or antibodies to deplete CD4 T-cells (α-CD4), CD8 T-cells (α-CD8) or both (α-CD4/ α-CD8) (a). Mice were weighed daily (b), monitored for survival (c) and body temperature monitored continuously using telemetry system (d). N = 16 for sham and 6-8 mice per other groups. Statistical comparisons performed using a two-way ANOVA with Dunnett's multiple comparisons test (b) or log-rank test with Bonferroni's correction for multiple comparisons (c). At day 5 p.i., viral loads in indicated tissues were quantified by qRT-PCR (e) and indicated statistical comparisons calculated using one-way ANOVA with Dunnett's multiple comparisons test. N = 6 per group. (b, d, e) Data shown as mean plus standard deviation. * P < 0.05, ** P < 0.01, *** P < 0.001.

Our data suggest that repNP and repGPC elicit distinct immune responses, primarily humoral and cellular responses, respectively. These data together with our efficacy data showing that repNP but not repGPC alone can confer protection against even high dose CCHFV challenge suggested that humoral immunity is the primary mediator of vaccine-induced protection. However, we sought to confirm this hypothesis through vaccination of mice lacking B-cells or depletion of T-cell populations at time of CCHFV challenge. To evaluate the contribution of humoral immunity groups of B-cell deficient mice (µMT) were vaccinated four weeks prior to CCHFV challenge with 1μg of repNP + repGPC in a prime-only regimen (Figure 6a). To evaluate the contribution of T-cells in vaccinated mice to control of CCHFV challenge, we treated mice with antibodies to deplete CD4^+^ T-cells (α-CD4), CD8^+^ T-cells (α-CD8), or both (α-CD4/α-CD8) just prior to CCHFV challenge on days -5 and -2 relative to CCHFV challenge. B- and T-cell deficient mice were compared against both sham and repNP + repGPC vaccinated WT mice. Depletion of T cells as well as verification of absent CCHFV-specific antibody in µMT mice was confirmed at day 0 (Supplemental Figure 5a-c). Notably, our ELISpot data showed that depletion of CD8^+^ but not CD4^+^ T-cells abolished IFNγ responses against the GPC peptide pool (Supplemental Figure 5c) suggesting that repRNA vaccination elicits primarily CD8^+^ T cells specific for the CCHFV GPC. Together these data confirmed that our approach successfully and specifically abolished vaccine-induced humoral or cellular immunity to CCHFV.

We then challenged the mice with CCHFV strain UG3010 as before. Vaccinated WT mice or vaccinated mice depleted of either CD4^+^ or CD8^+^ T-cells had no clinical disease and 100% of mice survived the CCHFV challenge (Figure 6b - d). In the α-CD4/α-CD8 group, two animals began losing weight between day 4 and 7 p.i. and exhibited a maximal weight loss of 14 & 19% on day 9 p.i. before beginning to recover after day 10 p.i. (Figure 6b). These data suggest that protection against CCHFV may be incomplete in mice lacking both CD4^+^ and CD8^+^ T-cells. In contrast, compared to WT vaccinated mice, all μMT mice exhibited clinical disease evidenced by significant (two-way ANOVA, P < 0.05) weight loss on days 4 – 9 p.i, significantly elevated body temperature on days 4 – 7 p.i. and 5 of 8 mice succumbed to disease (Figure 6b - d). However, compared to sham-vaccinated controls, clinical disease was delayed and survival significantly improved in vaccinated μMT mice (Figure 6b - d) indicating that vaccine-induced cellular immunity can contribute to control of the CCHFV infection. Consistent with the overt clinical disease and death in μMT mice, quantification of viral RNA at day 5 p.i. demonstrated that μMT mice failed to control the infection with significantly increased viral RNA loads compared to vaccinated WT mice and viral loads similar to sham-vaccinated mice (one-way ANOVA, P > 0.05) (Figure 6e). In the blood, mice depleted of T-cells had no significant increase in viral RNA loads suggesting that T-cells are dispensable for control of viremia (Figure 6e). However, in the liver and spleen we observed a trend towards increasing viral RNA loads compared to vaccinated WT mice (Figure 6e) suggesting that T-cells may contribute to control of viral replication in these tissues. Cumulatively, these studies demonstrate that repRNA-induced humoral immunity is essential for protection from clinical disease and control of viral replication while vaccine-induced cellular immunity alone is only partially protective against lethal disease in CCHFV-infected mice.

## Discussion

The lack of widely approved and available therapeutics for treatment of CCHFV and increasing range of CCHFV make development of a safe and efficacious vaccine a major goal for disease prevention. Our study reports a novel repRNA vaccine expressing the CCHFV NP and/or GPC. We found that a single immunization with as little as 50ng each of repNP and repGPC could induce robust and protective immunity against even a highly heterologous lethal CCHFV challenge. Our data also demonstrate that this protection is largely driven by repNP, encoding the more conserved CCHFV NP antigen. Alphavirus-based replicon vaccines have been previously evaluated for several viral pathogens^5^^,^^19^ and have many attractive features such as high protein expression, mimicry of an authentic viral infection, and single-round replication only. In our study we used a CNC-based delivery system^6^ to deliver the RNA *in vivo*. CNCs are highly stable, cationic emulsions that significantly enhance RNA stability and delivery via simple intramuscular immunizations.^6^ Additionally, this RNA vaccine and delivery system enable entirely cell-free production avoiding the issues of protein production, purification, safety and cost associated with systems such as DNA-plasmid, virus-vectored, subunit or virus-like particle platforms requiring bacterial, insect or mammalian cell culture. Using this platform, we have previously shown protection against Zika virus and SARS-CoV-2 in animal challenge models and the platform is currently being evaluated in human clinical trials for SARS-CoV-2 in India, Brazil, South Korea and the United States.^6^^,^^7^^,^^20^^,^^21^

In our studies we evaluated RNAs expressing either the CCHFV NP or CCHFV GPC. The majority of CCHFV vaccine approaches have focused on the use of the CCHFV GPC or glycoproteins^22^, ^23^, ^24^, ^25^, ^26^, ^27^, ^28^, ^29^ while some have utilized both NP and GPC.^30^^,^^31^ The viral glycoprotein Gc is the target of neutralizing antibodies^12^ and the GPC contains many protective neutralizing and non-neutralizing antibody epitopes.^12^^,^^32^^,^^33^ However, the GPC is the most genetically diverse segment of CCHFV and geographically distant strains of CCHFV have >25% amino-acid sequence divergence.^34^ The CCHFV NP is more conserved^34^ and vaccination with the NP antigen may confer protection against CCHFV on its own.^35^^,^^36^ Although much of the GPC diversity is located in the MLD with unclear function,^29^ escape from neutralizing antibodies by divergent strains is possible.^32^ Incomplete protection when the vaccine antigen and CCHFV challenge strain are mismatched has been reported for a DNA-based vaccine expressing only the GPC.^26^ A virus-like particle (VLP), DNA and recombinant vesicular stomatitis virus vaccine (rVSV) have shown heterologous protection^22^^,^^26^^,^^30^ while we have shown that previous infection with CCHFV could protect against heterologous re-infection^16^ demonstrating that heterologous protection is possible. Complete protection of vaccinated mice against a highly divergent CCHFV strain suggests our vaccine platform can confer broadly protective immunity against CCHFV.

The failure of repGPC-only vaccination to protect mice was surprising. We found that repGPC-only vaccination drove mainly a cellular immune response. Although our *in vitro* expression studies suggested that antigen expression driven by repNP was higher than repGPC, the significant T-cell responses directed against GPC in repGPC vaccinated animals suggests we achieved sufficient antigen production *in vivo* to elicit adaptive immune responses. Bunyavirus glycoproteins, including those of CCHFV, are targeted to the endoplasmic reticulum and Golgi networks^13^^,^^37^ and it is likely that repGPC drives similar localization and retention of the GPC antigen. In the absence of other CCHFV proteins to support proper protein trafficking or viral RNA for production of de novo virions, it is possible that repGPC-driven expression of the GPC is unable to achieve efficient presentation of Gn or Gc to B-cells and instead the intracellular retention promotes primarily cellular immunity.^38^ Future studies evaluating repRNAs expressing modified versions of the CCHFV glycoproteins are planned to investigate whether repRNA-driven expression can lead to humoral immunity against the glycoproteins. Our findings contrast with results from several other vaccines expressing the GPC or glycoproteins including modified vaccinia virus (MVA)-, DNA-, mRNA- and rVSV-based platforms that showed significant antibody responses in response to vaccination and protection against CCHFV challenge with the GPC or glycoprotein antigens alone.^24^^,^^27^^,^^39^^,^^40^ Our data in contrast to these previous reports indicate that the vaccine platform may significantly influence the protective efficacy of various CCHFV antigens.

While the modified vaccinia virus vaccine or mRNA-vaccine expressing the CCHFV glycoproteins (Gn and Gc) conferred 100% protection to lethally challenged mice, a DNA vaccine expressing the entire GPC only achieved >60% protection and a subunit vaccine expressing the ectodomains of the glycoproteins conferred no protection despite inducing neutralizing antibodies.^24^^,^^27^^,^^39^^,^^40^ However, the MVA and mRNA vaccines only evaluated homologous CCHFV challenge.^24^^,^^40^ Additionally, contrasting with our repNP results, an MVA expressing the CCHFV NP was immunogenic but failed to protect lethally infected mice.^41^ These distinct results suggest that the vaccine platform may influence whether vaccine-encoded NP or GPC is protective. We also cannot exclude the possibility that the divergence between vaccine antigen and CCHFV challenge strain in our studies may have enabled escape from otherwise protective T-cell responses as the Hoti-derived vaccine antigen and UG3010 challenge strain differ by 10% of amino acids in the region of GPC targeted by vaccine-induced cellular immunity.

Interestingly, we did not observe a significant effect of boosting on anti-CCHFV humoral and cellular immunity. The specific reason for failure of the boost to increase responses is unclear. Although previous studies have reported that anti-vector immunity can shorten antigen expression driven by alphavirus-replicons^42^ we have previously reported that mice immunized twice with the repRNA platform could respond to a third vaccination with a SARS-CoV-2-specific vaccine just four weeks after previous repRNA vaccination.^43^ These data suggest that anti-vector immunity is unlikely to completely abrogate immune responses to the vaccine. Instead, it is likely that timing of the boost is important for optimal efficacy of boosting with increasing time between prime- and boost-vaccinations leading to enhanced efficacy of boosting. Similar effects have been reported for SARS-CoV-2 RNA vaccines.^44^ Ongoing studies in mice and non-human primates with our CCHFV vaccine are evaluating longer periods between vaccinations.

Our study provides important insight into how CCHFV vaccines may confer protection against CCHFV challenge and addresses a significant gap in knowledge for CCHFV vaccine development. Although candidate vaccines against CCHFV have been found to elicit humoral and cellular immunity, the requirement of antibody and/or T-cells in control of the challenge remains largely untested. Interestingly, although both DNA and subunit vaccines induced neutralizing antibodies, this was not sufficient for complete protection in lethal CCHFV mouse models^27^^,^^39^ suggesting that neutralizing antibodies may be insufficient for protection against CCHFV. Similarly, some neutralizing Gc antibodies failed to protect mice from lethal CCHFV challenge in passive immunization experiments^12^ and incomplete protection was observed despite administration of a potently neutralizing antibody just 24 h after infection in lethally infected mice.^45^ Further, data from this study and others demonstrate that neutralizing antibodies are dispensable for vaccine-mediated protection.^40^^,^^46^ Thus, our data here demonstrate an essential role of CCHFV-specific antibody in protection from clinical disease and support a hypothesis that antibody effector function beyond neutralization is required for vaccine-mediated protection against CCHFV. This hypothesis is supported by findings that a single non-neutralizing antibody against the nonstructural GP38 protein encoded on the CCHFV GPC protected >90% of mice in a complement dependent manner.^47^ Our data extend these findings by suggesting that similarly non-neutralizing antibodies directed against the CCHFV NP can also confer protection. Indeed, repNP vaccination induced CCHFV-specific antibody predominantly of the IgG2c and IgG2b isotype, isotypes which possess potent Fc-effector activity.^48^ Taken together, these data suggest more complex mechanisms of repRNA-driven antibody-mediated protection than just neutralization of infectious CCHFV. However, our IFA data showed that repRNA-induced antibodies had little-to-no reactivity to surface-exposed antigen on virally infected cells arguing against mechanisms such as antibody-dependent cellular cytotoxicity or complement activation.^49^ Lastly, vaccinated μMT mice lacking any vaccine-induced humoral immunity had delayed clinical disease and improved survival, suggesting that the T-cell responses induced by repRNA vaccination can partially protect against clinical disease. Our findings significantly enhance our understanding of host protective immune mechanisms against CCHFV and our understanding on the roles of B- and T-cell responses to the CCHFV NP and GPC.

Our studies have some limitations that will require further studies to address. First, we did not evaluate durability of the immune response elicited by vaccination. An ideal vaccine for prevention of CCHF in endemic regions would elicit long-lasting protective immunity with minimal need for repeated vaccinations due to limited health care capacity. Second, although we report robust immunogenicity and protection in a lethal mouse model, further pre-clinical evaluations in non-human primate models^50^ and human clinical trials are needed. In recent evaluations of a replicating RNA vaccine for SARS-CoV-2 in human clinical trials, although well tolerated and immunogenic at low-doses, seroconversion was incomplete at the doses evaluated.^51^ However, our repRNA platform has shown immunogenicity against SARS-CoV-2 in mice, hamsters and non-human primates.^6^^,^^43^^,^^52^

In summary, we have demonstrated that a single low dose repRNA vaccination can induce both humoral and cellular immunity and confer protection against a lethal, highly heterologous CCHFV challenge in a stringent mouse model. Our findings define the role of humoral and cellular immunity in repRNA-mediated protection and significantly improve our understanding of how vaccines directed against CCHFV can confer protection. These findings will support continued development of our vaccine and others. The vaccine was well tolerated, easily administered, produced entirely synthetically and in conjunction with our robust efficacy data support continued pre-clinical and clinical development of the vaccine platform.

## Contributors

S.S.L., K.M.W., D.R., E.H., J.L., D.S. and D.W.H. performed the studies and data analyses. J.A., S.R. and J.E. generated vaccine materials. S.S.L., J.E., H.F. and D.W.H. designed the studies and verified data. H.F. secured funding. All authors have read and approved the final version of the manuscript.

## Data sharing statement

All data presented are available upon request.

## Declaration of interests

J.A. has equity interest in HDT Bio. J.E. has equity interest in HDT Bio, is a consultant for InBios and is a co-inventor on U.S. patent application no. 62/993,307 “Compositions and methods for delivery of RNA” pertaining to formulations for RNA delivery. DWH, JE and HF are inventors on U.S. patent application number 63/365,015 “Replicating RNA vaccine for Crimean-Congo hemorrhagic fever virus” regarding the repRNA for use against CCHFV.

## Appendix Supplementary materials

Image, application 1

Image, application 2
