# Supplementary material for: Replicating RNA vaccination elicits an unexpected immune response that efficiently protects mice against lethal Crimean-Congo hemorrhagic fever virus challenge
Source: eBioMedicine. 2022 Jul 27;82:104188. doi: 10.1016/j.ebiom.2022.104188 (PMC9335360; doi:10.1016/j.ebiom.2022.104188)
Supplement: Supplementary file 2 [file mmc2.pdf]

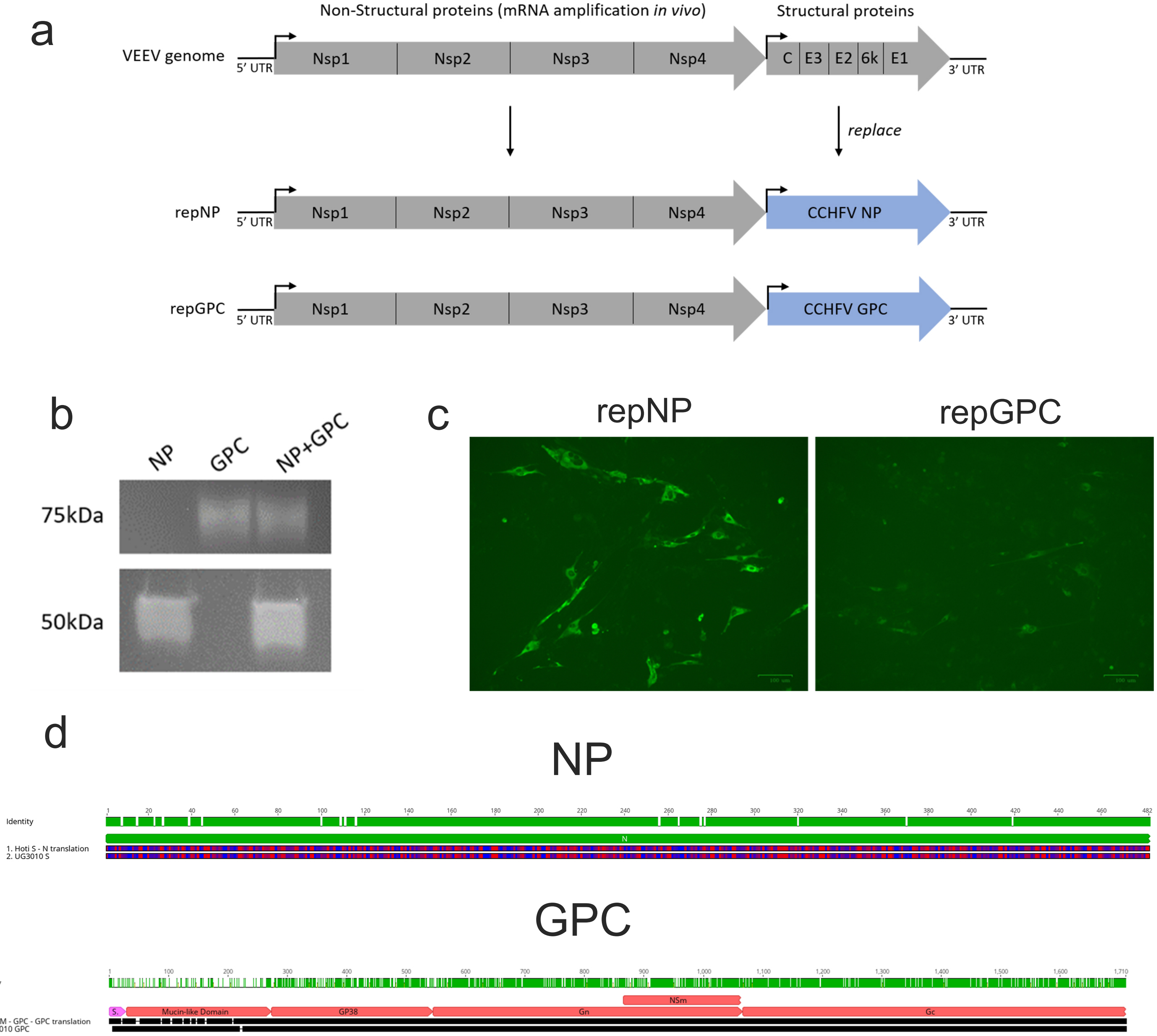

Supplemental Figure 1: (a) Generalized schematic of the repNP and repGPC RNAs. Western blot (b) or immunofluorescence (c) of BHK-21 cells transfected with indicated repRNAs with antibodies against the respective epitope tags of repNP and repGPC. (d) Amino acid alignments of vaccine encoded CCHFV strain Hoti NP and GPC to CCHFV challenge strain UG3010. Agreements indicated with green shading, disagreements indicated with no coloring. % Pairwise Identity: NP 96.5%, GPC 74.4%. Amino acid sequences aligned in Geneious Prime 2020.0.5 using Geneious Alignment.

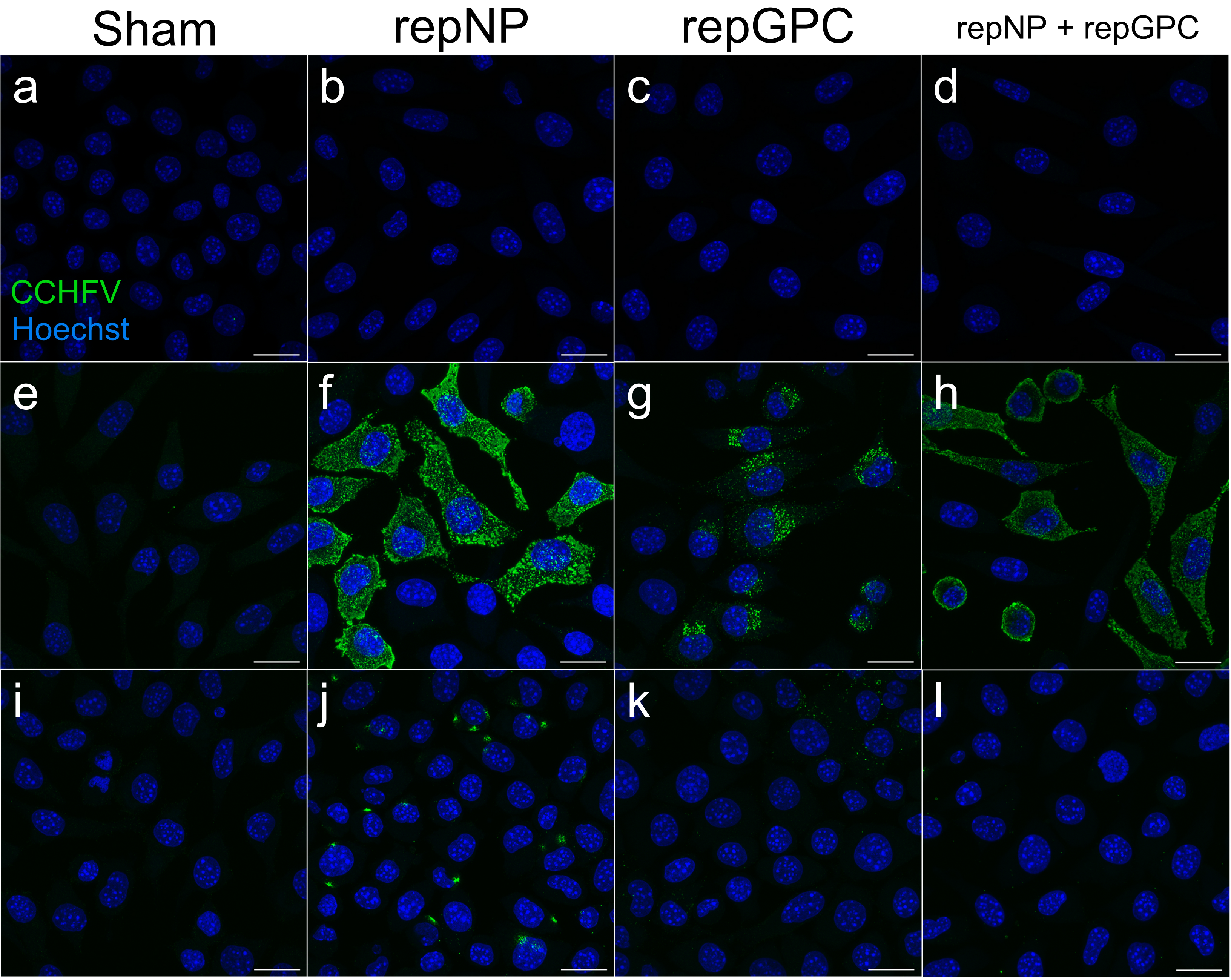

Suplemental Figure 2: Immunofluorescence of serum from repRNA-vaccinated mice. Serum was collected from vaccinated mice as in figure 1 and pooled. L929 cells were mock-infected (a-d) or infected with CCHFV strain Hoti at an MOI of 1 (e - l). At 24hpi, cells were fixed with paraformaldehyde and permeabilized with saponin (a-h) or unpermeabilized (i - l). Sera was applied at 1:500 and bound antibody detected with a goat anti-mouse IgG conjugated to AlexaFluor488. Cells were counterstained with Hoechst 33342. Samples were imaged using a Plan-Apochromat 63X/1.4 objective on a Zeiss laser scanning confocal microscope (LSM 880), driven by ZEN Black v 2.3 (Carl Zeiss Microscopy). All images were acquired with the same settings and processed identically for publication using Adobe Photoshop v 22.5.6. Scale bar represents 20μm. Representative fields shown.

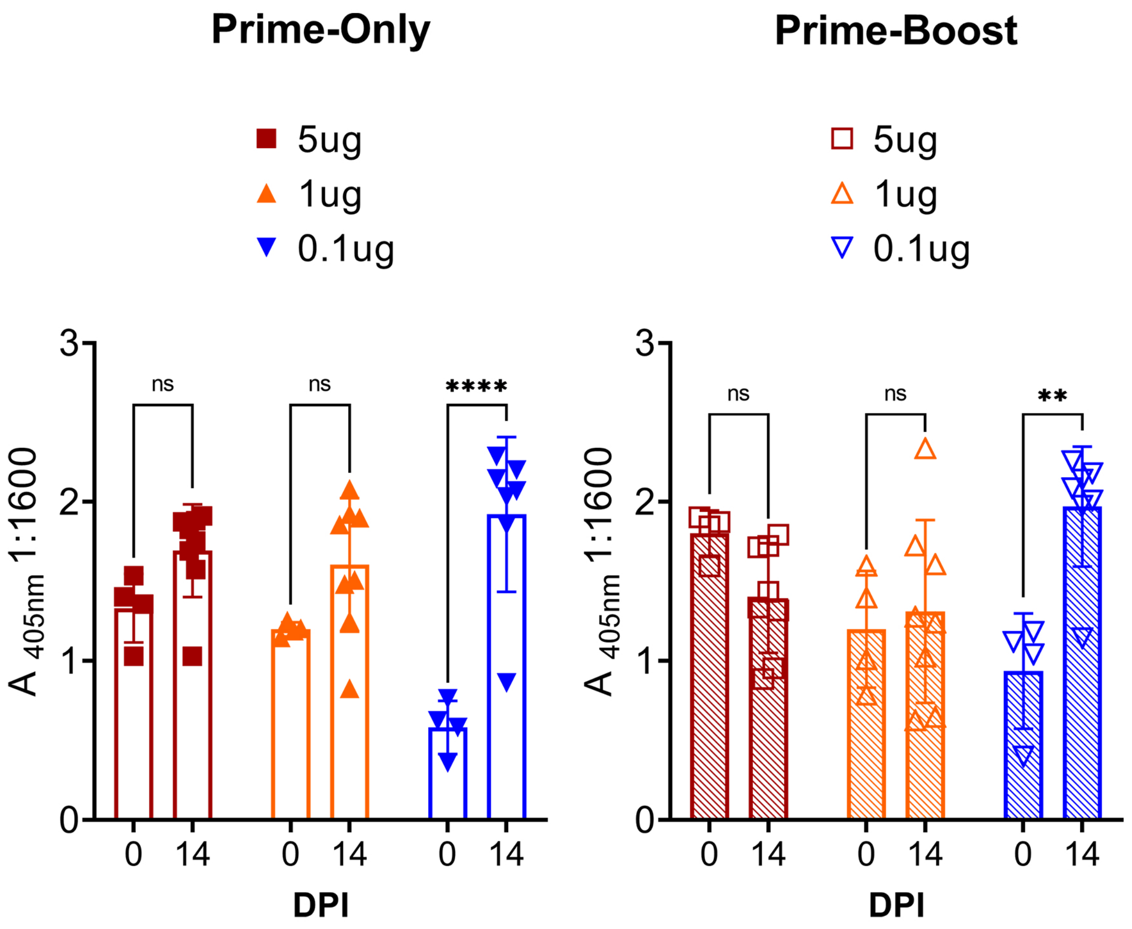

Supplemental Figure 3: Anamnestic antibody responses. At indicated time points post-infection, serum was collected from animals receiving indicated doses of repNP + repGPC following schedule as in figure 4. CCHFV-specific antibody responses were quantified by whole-virion ELISA. Day 0 data is duplicated from figure 4A and shown here for comparison. Indicated statistical comparisons calculated with two-way ANOVA with Sidak's multiple comparisons test. ns $P > 0.05$, ** $P < 0.01$, **** $P < 0.0001$. Data shown as mean plus standard deviation.

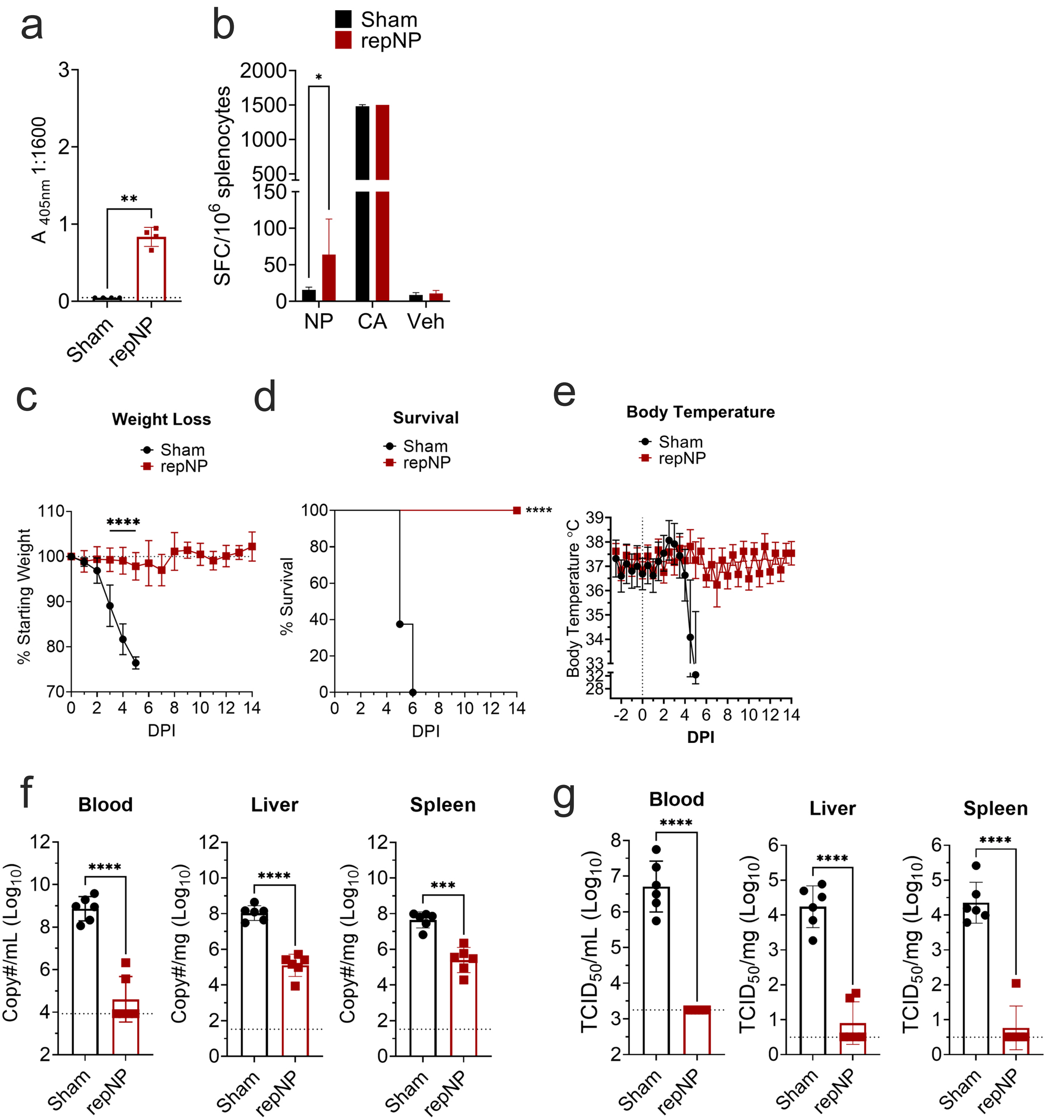

Supplemental Figure 4: Prime-only repNP vaccination protects against high-dose CCHFV challenge. On day -28 mice were vaccinated with 1μg of repNP via the IM route. On day 0, immune responses to vaccine were measured by ELISA (a) and IFN gama ELISpot (b). Mice were then treated with MAR1-5A3 to suppress type I IFN responses and challenged with 10,000 TCID50 of CCHFV strain UG3010 via the IP route. Mice were monitored daily and weight loss (c), survival (d) and body temperature (e) recorded. On day +4 PI, a group of mice was euthanized and viral loads in the blood, liver and spleen measured by qRT-PCR (f) or TCID50 assay (g). P values calculated using Welch's T-test (a, f, g), or two-WAY ANOVA with Sidak's multiple comparison test (b & c) and Log-rank test (d). ** $P < 0.01$, *** $P < 0.001$, **** $P < 0.0001$.

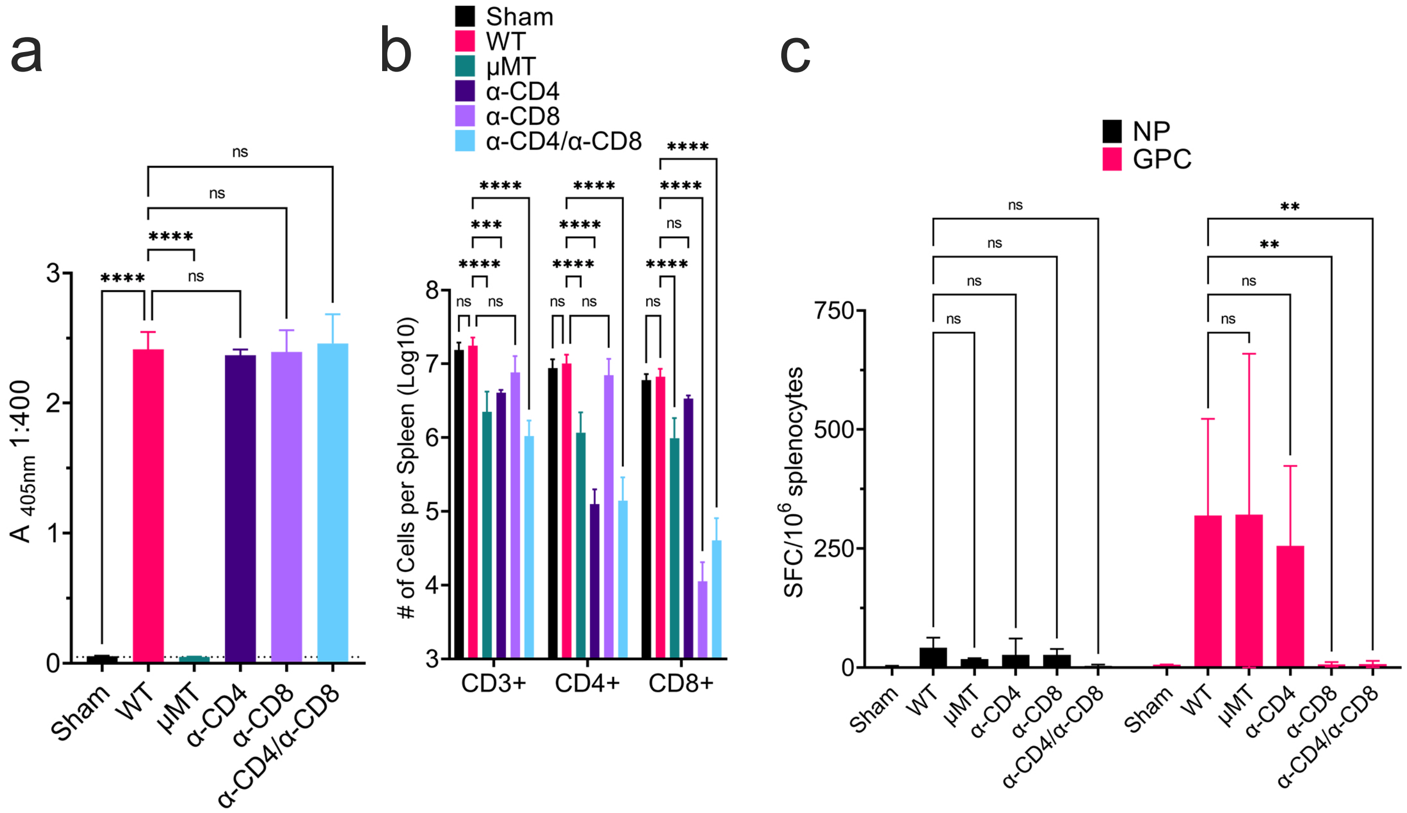

Supplemental Figure 5: On day 0,CCHFV-specific antibody was measured by whole virion ELISA (a). N = 4 mice per group. Dashed line indicates background absorbance of wells receiving no serum and indicated statistical comparisons calculated using one-way ANOVA with Dunnett's multiple comparison test. Efficacy and specificity of T-cell depletion in the spleen was measured by flow cytometry on day 0 (b). Cells were gated to exclude debris, doublets and non-viable cells. CD3+ T-cells were defined as CD45+CD3+B220- and CD4+ or CD8+ T-cells were defined as CD45+CD3+B220- and CD4+ or CD8+, respectively. N = 4 per group and indicated statistical comparisons calculated using two-way ANOVA with Dunnett's multiple comparison test. Effect of B-cell deficiency or T-cell depletion on CCHFV-specific T-cell responses was measured by IFNγ ELISpot against the immunodominant peptide pools identified for the CCHFV NP and GPC protein (c). N = 4 mice per group and indicated statistical comparisons calculated using two-way ANOVA with Dunnett's multiple comparison test. ns P > 0.05, * P <0.05, ** P < 0.01, *** P < 0.001, **** P < 0.0001. (a – c) Data shown as mean plus standard deviation.
